# The Midline Axon Crossing Decision Is Regulated through an Activity-Dependent Mechanism by the NMDA Receptor

**DOI:** 10.1523/ENEURO.0389-17.2018

**Published:** 2018-04-17

**Authors:** Jingxia Gao, Tamara J. Stevenson, Adam D. Douglass, Joshua P. Barrios, Joshua L. Bonkowsky

**Affiliations:** 1 Department of Pediatrics; 2 Department of Human Genetics; 3 Department of Neurobiology and Anatomy University of Utah School of Medicine Salt Lake City, Utah 84112

**Keywords:** activity, axon pathfinding, hypoxia, midline, NMDA receptor, zebrafish

## Abstract

Axon guidance in vertebrates is controlled by genetic cascades as well as by intrinsic activity-dependent refinement of connections. Midline axon crossing is one of the best studied pathfinding models and is fundamental to the establishment of bilaterally symmetric nervous systems. However, it is not known whether crossing requires intrinsic activity in axons, and what controls that activity. Further, a mechanism linking neuronal activity and gene expression has not been identified for axon pathfinding. Using embryonic zebrafish, we found that the NMDA receptor (NMDAR) NR1.1 subunit (*grin1a*) is expressed in commissural axons. Pharmacological inhibition of *grin1a*, hypoxia exposure reduction of *grin1a* expression, or CRISPR knock-down of *grin1a* leads to defects in midline crossing. Inhibition of neuronal activity phenocopies the effects of *grin1a* loss on midline crossing. By combining pharmacological inhibition of the NMDAR with optogenetic stimulation to precisely restore neuronal activity, we observed rescue of midline crossing. This suggests that the NMDAR controls pathfinding by an activity-dependent mechanism. We further show that the NMDAR may act, via modulating activity, on the transcription factor *arxa* (mammalian *Arx*), a known regulator of midline pathfinding. These findings uncover a novel role for the NMDAR in controlling activity to regulate commissural pathfinding and identify *arxa* as a key link between the genetic and activity-dependent regulation of midline axon guidance.

## Significance Statement

While intrinsic neuronal activity is involved in refinement of axon connections, its role in pathfinding decisions is poorly understood. We found that midline axon crossing is regulated by the NMDA receptor (NMDAR). The NMDAR is expressed on axons that cross the midline, and inhibition or knock-down of the NDMDAR led to fewer axons crossing. Precise optogenetic stimulation of neurons rescued the effects of NMDAR blockade, demonstrating that the NMDAR acts by an activity-dependent mechanism. In turn, the NMDAR affects expression of *arxa*, an important gene for brain development and that is associated with several human neurologic diseases. These results show a critical role for the NMDAR in early axon guidance decisions by control of neuronal activity.

## Introduction

A key common feature in the CNS of invertebrates and vertebrates is the establishment of a bilaterally symmetric nervous system, which in turn requires forming connections between the two sides. Most axons cross the midline once and do not cross again, while a smaller percentage remain ipsilateral and never cross (Garbe and Bashaw, 2004). Genetic regulation of the midline crossing decision has been intensely studied since the 1990s, leading to the identification of canonical axon guidance gene families, including the Robos, Slits, Netrins, Dccs, Uncs, Derailed, Frizzleds, and Wnts (Dickson, 2002). Abnormalities of commissural axon tracts, caused by genetic disorders or premature birth, are associated with a variety of neurodevelopmental disabilities ranging from isolated intellectual impairment to autism spectrum disorder (Hardan et al., 2009; Izzi and Charron, 2011; Marsh et al., 2017).

The standard model of pathfinding in vertebrates consists of two parts: an early, “hard-wired” genetically controlled phase; and later, neuronal activity refinement of connections, particularly of synapses. A defined genetic cascade, culminating particularly in the expression of cell-surface receptors and ligands, guides early pathfinding; whereas intrinsic neuronal activity appears to be regulated separately for later aspects of connectivity development (Bixby and Harris, 1991; Katz and Shatz, 1996; Tessier-Lavigne and Goodman, 1996; Yamamoto and López-Bendito, 2012; Kutsarova et al., 2017). However, recent studies have shown an early role for neuronal activity in the development of normal connectivity (Hanson and Landmesser, 2004; Spitzer, 2006). For example, the terminal projection determination of retinofugal, retinotectal, and commissural axons require neuronal activity (Schmidt et al., 2000; Mizuno et al., 2007, 2010, Dhande et al., 2011), and interhemispheric coordinated activity is necessary for final targeting of axon projections once they have crossed the midline (Suárez et al., 2014). Whether there is a mechanism linking genetic regulation and neuronal activity, or whether the two act independently, is uncertain, with conflicting evidence for both possibilities (Hanson and Landmesser, 2004; Nicol et al., 2007; Cang et al., 2008; Benjumeda et al., 2013).

Thus, key unresolved questions remain regarding the role of activity and commissural axon projections. First, it is not known whether the choice for midline crossing requires intrinsic activity in the axons. If activity is required for the midline crossing choice, what controls their activity? Second, in early development, during the period classically considered to be primarily hard-wired and genetically regulated, is there a mechanism linking neuronal activity and gene expression?

To address these questions, we tested the role of activity in the midline crossing choice using embryonic zebrafish (*Danio rerio*) to visualize guidance decisions. The embryonic zebrafish is well suited for analysis of axon guidance: it is transparent, facilitating visualization of pathfinding; and experimentally accessible to pharmacological, optogenetic, and genetic manipulation. To explore the requirement for activity, we examined the role of the NMDAR for three reasons: first, because of its known role in axon refinement and branch maturation (Ben Fredj et al., 2010); second, because the NMDAR is widely expressed in the early zebrafish embryo CNS (Cox et al., 2005); and third, prior work has shown that NMDARs are present in axon growth cones (Wang et al., 2011; Gill et al., 2015), but their involvement in axon guidance has not been studied and no evaluation of their functional role has been performed.

Here, we report that the NMDAR is required for midline axon crossing. We found that the NR1.1 subunit of the NMDAR (*grin1a*) is expressed in a population of commissural axons. Pharmacological inhibition or CRISPR knock-down of *grin1* leads to defects in midline crossing. Inhibition of neuronal activity phenocopies the effects of *grin1* loss. Finally, we use optogenetic stimulation to precisely rescue neuronal activity in pharmacologically treated embryos, demonstrating that the NMDAR’s role in pathfinding is mediated by neuronal activity. We find that the NMDAR may act, via modulating activity, on the transcription factor *arxa* (mammalian *Arx*), a known regulator of midline pathfinding. Thus, our study demonstrates a novel role for the NMDAR in early commissural pathfinding and demonstrates an early requirement for neuronal activity in midline axon guidance.

## Materials and Methods

### Ethics statement

Zebrafish experiments were performed with supervision and in accordance of guidelines from the University of Utah Institutional Animal Care and Use Committee, regulated by federal law (the Animal Welfare Act and Public Health Services Regulation Act) by the United States Department of Agriculture and the Office of Laboratory Animal Welfare at the National Institutes of Health, and accredited by the Association for Assessment and Accreditation of Laboratory Care International.

### Fish stocks and husbandry

Adult fish were bred according to standard methods and staged by time and morphology (Kimmel et al., 1995). Embryos were raised at 28.5°C in E3 embryo medium with methylene blue and embryos beyond 24 h postfertilization (hpf) were treated with phenylthiourea (PTU) to prevent pigmentation.

Transgenic fish lines and alleles used in this study were the following: Tg(*th2:Gal4-VP16*)^zd202^ (McPherson et al., 2016), Tg(*foxP2-enhancerA.2:egfp-caax*)^zc69^; Tg(*cmcl2:EGFP; foxP2-enhancerA.2:Gal4-VP16_413-470_*)^zc72^ (Stevenson et al., 2012); and Tg(*UAS:ChR2-YFP*)^a144^ (Lacoste et al., 2015). Lines are available from the Zebrafish International Resource Center or on request.

### Hypoxia conditions

To induce hypoxia, embryonic zebrafish were placed in a sealed Plexiglas chamber connected via a controller that monitored and adjusted nitrogen gas flow to a desired pO_2_ set point (Biospherix, Inc.; Stevenson et al., 2012). Embryos were incubated in 1% O_2_ from 24 to 36 hpf. Morphologic staging was used to determine age at fixation for analysis.

### Scoring tract of the commissure of the posterior tuberculum (TCPTc) C/L ratios

Evaluation of the TCPTc axon midline crossing defects was determined by the following approach. Briefly, a confocal z-stack was taken of the region using identical confocal settings (20× objective, imaging speed: 8 μs/pixel, laser power: 30%, gain: 1.25%, offset: 5%). We used ImageJ to generate a maximum intensity projection of 11 slices (step size: 2 μm); then subtracted the background fluorescence by using a rolling ball radius of 50 pixels. We measured the total fluorescence intensity of a rectangular area (10 × 30 μm) placed over the commissure of TCPT pathway, or of the longitudinal tract before its decussation into the commissure. A ratio of commissural versus longitudinal axon intensity (C/L ratio) was calculated (Stevenson et al., 2012; Xing et al., 2015). Since experimental variation was noted, results were only compared between experiments performed on the same day. At least 10 embryos were imaged for each treatment.

### Immunohistochemistry and *in situ* hybridization

For *in situ* hybridization and immunohistochemistry, embryos were fixed in 4% paraformaldehyde (PFA) in PBS overnight at 4°C, washed briefly in PBS with 0.1% Tween 20, dehydrated stepwise in methanol (25%, 50%, 75%, 100%), and stored in 100% MeOH at −20°C until use.

Immunohistochemistry was performed as described previously (Bonkowsky et al., 2008). Following fixation and dehydration in methanol, embryos were rehydrated, permeabilized using proteinase K [10 μg/ml in PBS with 0.1% Tween 20 (PBST)] at 28°C for 60 minutes (min) (8 min for 24 hpf, 20 min for 36 hpf, and 30 min for 48 hpf) without rocking, washed twice in PBST for 5 min then re-fixed for 15 min. Embryos were then washed in PBST, blocked in PBST/1% DMSO/2% BSA/5% normal goat serum (NGS), and then incubated overnight in a primary antibody solution diluted in PBST/1% DMSO/2% BSA/2% NGS at 4°C. The next day embryos were washed in PBST/1% DMSO/1% NGS for a minimum of 6 h, followed by incubation overnight with secondary antibodies, and washed the following day.

Antibodies used were the following: chicken polyclonal green fluorescent protein (GFP) antibody 1:1000 (Aves Lab, GFP-1020), rabbit polyclonal anti-VGluT1 1:100 (Abcam, ab77822), mouse anti-acetylated tubulin 1:250 (Developmental Studies Hybridoma Bank, 6G7), mouse monoclonal anti-GluN1 1:50 (Synapse System, #114 011), goat polyclonal anti-EphrinB2a 1:20 (R&D Systems, AF496), Alexa Fluor 488-conjugated goat anti-chicken 1:400 (Invitrogen, A-11039), Cy3 conjugated anti-rabbit 1:400 (Invitrogen, A10520), and Alexa Fluor 555-conjugated donkey anti-mouse 1:400 (Invitrogen, A-31570).

Whole-mount in situ hybridization for *NR1.1* was performed using an anti-sense probe (Cox et al., 2005) following our previous protocol (Bonkowsky and Chien, 2005).

For *in situ* hybridization combined with immunohistochemistry, after *in situ* hybridization was completed, embryos were transferred sequentially through 5%, 15%, and 30% sucrose in PBS. Embryos were then embedded in optimal cutting temperature compound (OCT; Tissue-TeK) using a dry ice-ethanol bath and stored at −80°C overnight. Fourteen-micrometer sections were cut for immunostaining. Sections were boiled for 20 min with citrate buffer (10 mM sodium citrate and 0.05% Tween 20; pH 6.0) for antigen retrieval, and immunostaining was performed as described above.

### RNA extraction, reverse transcription, and quantitative real-time polymerase chain reaction (qRT-PCR)

The 24 hpf whole embryos or the head tissue of 48 hpf embryos were homogenized in TRIzol reagent (Ambion) and total RNA was extracted and purified with the RNeasy mini kit (QIAGEN). An aliquot of each extract was used for spectrophotometry to determine RNA quality and concentration. cDNA was synthesized from total RNA (2 μg; 20-μl final reaction volume) with oligo(dT) priming using SuperScript III reverse transcriptase (ThermoFisher) according to the manufacturer's instructions. The resulting cDNA was diluted 4–20 times for the real time quantitative PCR. Samples were prepared with three independent biological replicates. RT-PCR was performed on an ABI prism 7900 HT instrument (Applied System) with a SYBR green fluorescence label. qRT-PCR for *grin1a* and *grin1b* was performed on 24 and 48 hpf embryos. Reactions were run with the following conditions: 95°C for 10 min and 40 cycles of 95°C 20 s (s)/60°C 20 s/72°C 40 s. Each reaction was performed in triplicate and the mean of replicates was calculated; results were normalized to the mRNA level of each gene in normoxia embryos at 24 and 48 hpf with elongation factor 1α (*elf1a*) transcript levels as a control using the 2^−ΔΔCT^ method (Livak et al., 2001). For statistical analysis, Student’s *t* test was used to compare between normoxia and hypoxia groups. Primers for *grin1a* were forward 5’-AAGCCAACTACGCTGGAAGG-3’ and reverse 5’-CAATTGGGCGTCCTGGGAT-3’; for *grin1b* were forward 5’-TTGACAACAAGCGAGGACCC-3’ and reverse 5’-CGTCTTCACTTGCAGACAGGA-3’; and for *elf1a* were forward 5’-CTTCTCAGGCTGACTGTGC-3’ and reverse 5’-CCGCTAGCATTACCCTCC-3’ (McCurley et al., 2008).

### *Grin1a* and *grin1b* CRISPR/Cas9

One-cell stage embryos were injected with 400 pg of cas9 nuclease 3NLS (Integrated DNA Technologies) and 200 pg each of *grin1a* and *grin1b* sgRNA. sgRNAs were targeted to the following sequences in *grin1a*: GCAGGAGCAGGAGAACAACG and in *grin1b*: GATGGCACTCTCCGTGTGCG. sgRNA synthesis was followed the protocol as described previously. Embryos were collected at 24 hpf for mutation analysis using PCR and high-resolution melt analysis (HRMA). Genomic DNA was prepared from 24 hpf embryos and PCR was performed using the following primers: *grin1a* forward 5’-ATGCGTCTGCTTCTGCTGGC-3’ and reverse 5’-CTTCTGGCTCAGGACAGCCC-3’, and *grin1b* forward 5’-GTCACCCATAAGCCAAACGCC-3’ and reverse 5’-GACCTGAACAGACTGTTTTACCTGG-3’. HRMA was performed on a LightScanner-96 instrument (Idaho Technology) from 65°C to 95°C with a temperature transition rate of 0.1°C/s. For sequencing the targeted regions in F0 founders, PCR amplicons were cloned into pCR4-TOPO TA vector (Invitrogen). Plasmids were prepared from single colonies and Sanger sequenced to identify the mutation(s).

### Microscopy and image analysis

Image acquisition and analysis were performed as described previously (Stevenson et al., 2012). Immunostained embryos were transferred sequentially into 20%, 50%, and 80% glycerol in PBST, mounted in a glass slide with a #0 coverslip placed over a well made using electrical tape, and imaged on a confocal microscope (Olympus FV-1000). Confocal stacks were projected in ImageJ and composed with Adobe Illustrator.

To compare fluorescence intensity of neuron cell bodies in control versus APV-treated embryos, we took confocal images with identical settings. Following image acquisition, we generated maximal intensity z-stack images and subtracted background using the rolling ball (100) setting in ImageJ. For each hemi-telencephalon we measured the fluorescence intensity using a square of set size which included all the cell bodies. We calculated total fluorescence intensity = area*mean intensity, and then averaged the hemi-telencephalon values for each single embryo.

### Drug treatment

The drugs used in this study were 500 μM D-APV (Tocris), 500 μM MK-801 (Tocris) and 500 μM NMDA (Tocris). Embryos were manually dechorionated at 24 hpf before drug exposure. PTU-containing E3 media were mixed with the respective drugs unless otherwise noted. Embryos were incubated with the drugs from 24 to 72 hpf and were fixed at 72 hpf.

For NMDA experiments, magnesium-free E3 medium (5 mM NaCl, 0.17 mM KCl, 0.33 mM CaCl_2_, and 0.00002% methyl blue) were used. The embryos were incubated in PTU-containing magnesium-free E3 medium mixed with NMDA in normoxic or hypoxic conditions.

### Western blot analysis

Thirty embryos (72 hpf) were deyolked by incubating in deyolking buffer (65 mM NaCl, 1.7 mM KCl and 1.5 mM NaHCO_3_) for 10 min and triturating vigorously. Protein was extracted by grinding embryos with pestle in a 60 μl of lysis buffer [150 mM NaCl, 20 mM Tris-HCl (pH 7.5), 1 mM EDTA, 1% NP-40, and 1% Triton X-100 and protease inhibitor; 1:100, Sigma] on ice. The extract was centrifuged for 10 min at 4°C, and the supernatants were transferred to a new tube. The protein extract was then mixed with an equal amount of 2× Laemmli buffer [20% glycerol, 4% SDS, 0.1% bromophenol blue, 0.125 M Tris (pH 6.8), and 2.5% β-mercaptoethanol] and heated at 95°C for 10 min. Samples were stored at −20°C until use.

A total of 40 μl of samples was then run on a 4–20% gradient gel (Miniprotean TGX Gel Bio-Rad), and electro-blotted onto a PVDF membrane. After blocking with 3% non-fat milk in TBS (50 mM Tris, 0.138 M NaCl, and 2.7 mM KCl; pH 8.0) for 30 min with agitation, the membrane was split, and then incubated in mouse monoclonal anti-GluN1 antibody (Synapse System, #114 011, 1:100), anti-EfnB2a (R&D Systems, AF1088, 1:20), or rabbit anti-β-actin (Abcam, ab8227, 1:1000) at 4°C overnight; washed in TBS; and then incubated in HRP-anti-mouse (Cell Signaling, #7076, 1:1000) or HRP-anti-rabbit (Cell Signaling, #7074, 1:5000) for 1 h at room temperature. Following incubation with secondary antibody, the membrane was washed extensively in TBS containing 0.05% Tween 20 and then subjected to chemiluminescent detection (WesternBright Quantum, Advansta). The intensity of specific bands was quantitated using ImageJ in three separate experimental replicates.

### Optogenetics experiment and genotyping of the embryos

For optogenetics experiments, embryos were obtained from matings of Tg(*foxP2:egfp-caax*); Tg(*cmcl2:GFP; foxP2:gal4-VP16*) fish crossed with Tg(*th2:gal4-VP16*); and Tg(*UAS:ChR2-YFP*) fish. Activation from the Tg(*UAS:ChR2-YFP*) line was demonstrated previously (McPherson et al., 2016). The 24 hpf embryos were dechorionated and stimulated with 470-nm light exposure from a high-power LED at 0.6 mW/mm^2^, distributed uniformly across a 35-mm Petri dish with a condenser lens. The embryos were stimulated for 48 h, consisting of a 5-s exposure and intervening 300 s off, from 24 to 72 hpf. The embryos were fixed at 72 hpf and subjected to immunohistochemistry analysis. After completion of immunohistochemistry, embryos were mounted and fin-clipped correspondingly, and the genotype was determined by PCR for the presence of *UAS:ChR2-YFP* (which is indistinguishable in the cross from EGFP-caax) and for the absence of *th2:Gal4* (which is not apparent until ∼4 dpf). The primers for Tg(*UAS:ChR2-YFP*) were forward 5’-GATGGATTGAATCTCGCGGC-3’ and reverse 5’-GTTGCTCAGGCGGATAAGGA-3’ and for Tg(*th2:Gal4-VP16*) were forward 5’-TAAATTGCGGGGATGTGGCC-3’ and reverse 5’-GGTTTTTCTTTGGAGCACTTGAGC-3’.

### Statistical analysis

Analysis was done blinded to experimental condition and/or genotype. Experiments were performed before assignment of sex in zebrafish, during embryonic and larval stages. C/L ratios and Western blotting intensities were tested for significant differences using Student’s *t* test. qRT-PCR *p* values were adjusted using Benjamini–Hochberg false discovery rate. For all analyses, *p* < 0.05 was considered significant. Analyses were conducted with Prism 6 (GraphPad).

## Results

### The NMDAR subunit NR1.1 (*grin1a*) is expressed in crossing axons

To study the role of NMDAR in midline axon pathfinding, we used the transgenic line *Tg(foxP2-enhancerA.2:egfp-caax)*, which expresses membrane-bound GFP in a subset of neurons in the telencephalon (Stevenson et al., 2012). Tg*(foxP2-enhancerA.2:egfp-caax)* neurons project axons in the anterior commissure, longitudinal pathways, and the diencephalic TCPTc (Wilson et al., 1990). The TCPTc axons start to extend by 24 hpf; by 36–48 hpf, axons begin to cross the midline; and most axons have crossed by 72 hpf (Stevenson et al., 2012; Xing et al., 2015). The Tg*(foxP2-enhancerA.2:egfp-caax)* line is particularly useful for visualizing midline crossing because it labels a subset of telencephalic neurons and axons (∼500–1000 in each hemi-telencephalon).

**Figure 1.**
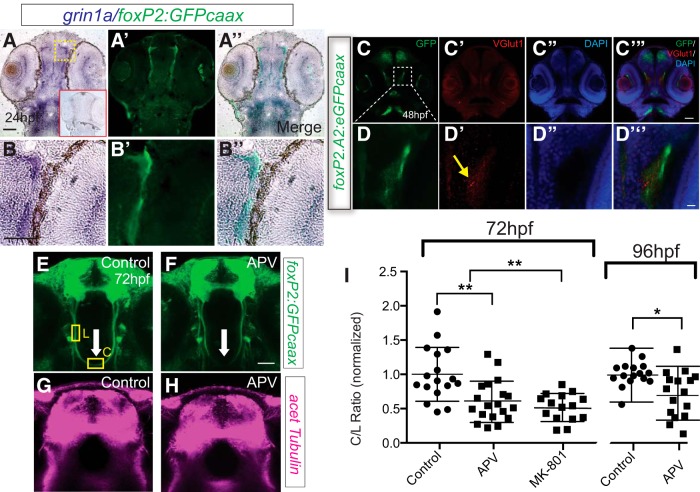
*foxP2-enhancerA.2:egfp-caax* axons coexpress and are adjacent to a glutamate source (Vglut1 expression). ***A–B’’***, Sections of 24 hpf embryos, α-GFP immunohistochemistry, and *grin1a in situ*, rostral top; scale bar = 50 µm, 25 μm (***B–B’’***). Red boxed inset in first panel shows embryo stained with *grin1a* sense probe. Tg(*foxP2-enhancerA.2:egfp-caax*) embryo has coexpression of GFP and *grin1a* in TCPT axons. ***C–D’’***, Confocal whole-mount images of 36-hpf embryos, α-GFP immunohistochemistry, α-Vglut1 immunohistochemistry, and DAPI nuclear stain, rostral top; scale bar = 50 µm, 10 μm (***D–D’’***). TCPT axons pass adjacent to glutamatergic neurons. ***E–G***, NMDAR blockade reduces midline crossing. ***E–F***, Confocal image of Tg(*foxP2-enhancerA.2:egfp-caax*) embryos, showing where measurements were made for the intensity of commissural (C) and longitudinal (L) axon tracts (for details, see Materials and Methods). Maximum intensity z-stack projections, α-GFP immunohistochemistry, rostral top; scale bar = 50 µm. TCPTc midline axon crossing is disrupted (arrow) when treated with APV (***F***) compared to control (***E***). ***G***, ***H***, Confocal images of the telencephalon show no difference in overall axon projection patterns and densities of APV-treated compared to control embryos. Maximum intensity z-stack projections, α-acetylated tubulin immunohistochemistry, rostral top. ***I***, Scatterplot C/L quantification results performed at 72 or 96 hpf; *p < 0.05; **p < 0.01; ANOVA with *post hoc* Tukey’s HSD or Student’s *t* test. Data are in Extended Data Figures 1-1, 1-2.

We examined expression of the NMDAR subunit GluN1 (*grin1a)*. We found that *grin1a* is expressed in TCPTc axons at 24 hpf, as they extend and prepare to cross the midline (Fig. 1*A–B’’*). Next, we evaluated whether there is a source of glutamate that could serve as a ligand for the NMDAR. We observed that TCPTc axons pass near regions expressing Vglut1 (*slc17a7a*), the vesicular glutamate transporter expressed on neurons that release glutamate (Takamori et al., 2001; Fig. 1*C–D’’’*). These findings show that the NMDAR is expressed in TCPT commissural axons, and that there is a glutamate source for the TCPT axons as they prepare to cross the midline.

### Inhibition of NMDAR decreases midline crossing

We used pharmacological inhibition of the NMDAR to test its necessity for midline crossing. To quantify the extent of the crossing defect, we measured the ratio of commissural to longitudinal axon crossing (the C/L ratio) in the TCPT commissural axons of Tg*(foxP2-enhancerA.2:egfp-caax)* using our previous methods (Stevenson et al., 2012; Xing et al., 2015; Fig. 1*E*,*F*). Using APV (2-amino-5-phosphonopentanoic acid), a competitive NMDAR antagonist that blocks the glutamate binding site; or MK-801 (dizocilpine), an uncompetitive NMDAR antagonist that is use- and voltage-dependent; we found a significant decrease in crossing axons at 72 hpf (one-way ANOVA; control, APV, MK-801; *n* = 32, *n* = 19, *n* = 15; mean C/L 1.0, 0.61, 0.59; SD 0.43, 0.30, 0.23; *F*_(2,63)_ = 10.3, *p* < 0.001; *post hoc* Tukey’s HSD control versus APV, *p* < 0.01; control vs MK-801, *p* < 0.01; Fig. 1*E*,*F*,*I*; Extended Data Figs. 1-1, 1-2). Importantly, the decrease in crossing axons was not simply due to a delay in development; the C/L ratio was also still significantly different at 96 hpf, >24 h after the end of drug exposure (*n* = 15 each, control and APV, mean C/L 1.0, 0.76; SD 0.20, 0.30; *p* < 0.05, Student’s *t* test; Fig. 1*I*). Also, the effect of APV was not due to an overall change in axon outgrowth. Visualization with the pan-axonal anti-acetylated tubulin antibody showed no difference between control and APV-treated embryos in terms of overall axon staining (Fig. 1*G*,*H*).

Treatment with APV or MK-801 did not adversely affect embryo morphology or development. Further, the treatment did not change properties of the reporting transgene: we found that APV did not change fluorescence of the *foxP2.A.2:egfp-caax* transgene line. We measured intensities of the neuron cell bodies and found that in control compared to APV-treated, average total fluorescence pixel intensity was 1.11 × 10^6^ versus 1.18 × 10^6^ (*n* = 8 each, both hemi-telencephalon cell groups counted, SD 5.6 × 10^4^ vs 3.3 × 10^5^, Student’s *t* test, *p* = 0.62). These results suggest that NMDAR function is necessary for normal midline crossing.

### NMDAR interacts genetically with known modulators of midline crossing

**Figure 2.**
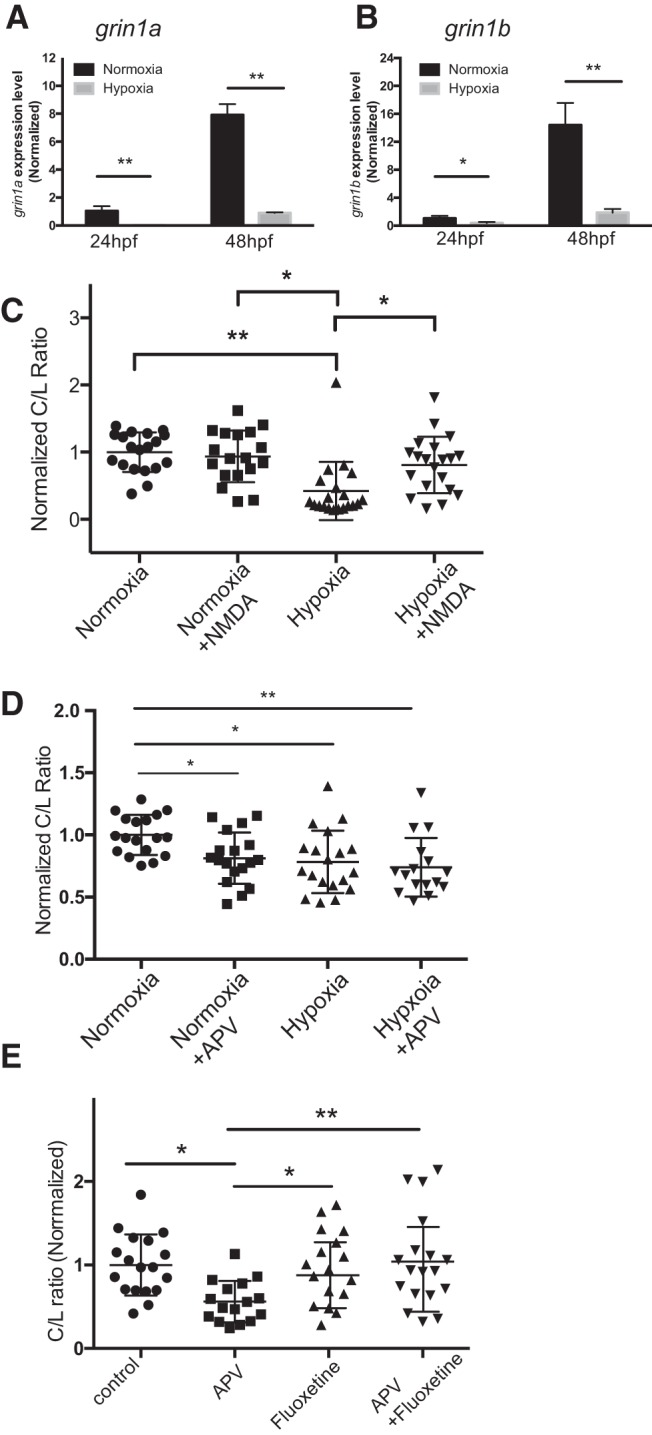
The NMDAR interacts with other known mechanisms to control midline crossing. ***A***, ***B***, Hypoxia causes reductions of *grin1a* and *grin1b* subunit expression at 24 and 48 hpf, during TCPT midline crossing. qRT-PCR, triplicate experiments, normalized to *elf1a*. Error bars, SEM; *p < 0.05; **p < 0.01. ***C–E***, Scatterplot C/L results for TCPT crossing, ns, not significant; *p < 0.05; **p < 0.01; ANOVA with *post hoc* Tukey’s HSD. ***C***, Hypoxic reduction of midline crossing is rescued by addition of NMDA. ***D***, Blockade of NMDAR does not worsen hypoxia impairment of midline crossing. ***E***, Increased serotonin signaling rescues midline crossing defect from NMDAR blockade.

Previous work has shown that the midline crossing decision can be modulated by exogenous signaling due to potential environmental factors, including hypoxia or exposure to serotonergic compounds (Pocock and Hobert, 2008; Bonnin et al., 2011; Stevenson et al., 2012; Xing et al., 2015). Since we had observed that pharmacological inhibition of NMDAR caused a similar midline axon phenotype, this suggested a potential shared or interacting mechanism. To explore this, we tested whether the NMDAR interacted with hypoxia and with serotonin signaling. It has been shown that hypoxia reduces TCPTc crossing (Stevenson et al., 2012), and hypoxia is known to affect NMDAR activity and expression (Kobayashi and Millhorn, 2000; Mishra et al., 2001). We assessed *grin1a* and *grin1b*, the paralogs of the GluN1 subunit of the NMDAR. First, we found that hypoxia reduced expression of *grin1a* and *grin1b* (Fig. 2*A*,*B*). Addition of exogenous NMDA rescued the effects of hypoxia on TCPTc crossing (Fig. 2*C*; one-way ANOVA; normoxia, normoxia with NMDA, hypoxia, hypoxia with NMDA; *n* = 18 each; mean C/L 1.0, 0.93, 0.42, 0.81; SD 0.29, 0.39, 0.43, 0.42; *F*_(3,73)_ = 8,7, *p* < 0.001; *post hoc* Tukey’s HSD normoxia vs hypoxia, *p* < 0.01; hypoxia vs hypoxia with APV, *p* < 0.05; normoxia with APV vs hypoxia, *p* < 0.05).

In contrast, blockade with APV did not worsen the phenotype caused by hypoxia (Fig. 2*D*; one-way ANOVA; normoxia, normoxia with APV, hypoxia, hypoxia with APV; *n* = 16 each; mean C/L 1.0, 0.81, 0.78, 0.74; SD 0.16, 0.21, 0.25, 0.24; *F*_(3,67)_ = 5.0, *p* < 0.01; *post hoc* Tukey’s HSD normoxia vs normoxia with APV, *p* < 0.05; normoxia vs hypoxia, *p* < 0.05; normoxia vs hypoxia with APV, *p* < 0.01). We also explored the interaction between serotonergic signaling and NMDAR’s effects, because it has been shown that the serotonin receptor *htr2a* is necessary for TCPT crossing and is a mediator of hypoxia’s effect (Xing et al., 2015). We found that increasing available serotonin by using fluoxetine, a serotonin re-uptake inhibitor, restored a normal ratio of midline crossing in APV-treated embryos (Fig. 2*E*; one-way ANOVA; control, APV, fluoxetine, fluoxetine with APV; *n* = 16 each; mean C/L 1.0, 0.56, 0.96, 1.0; SD 0.16, 0.21, 0.25, 0.24; *F*_(3,66)_ = 4.6, *p* < 0.01; *post hoc* Tukey’s HSD, control vs APV, *p* < 0.05; APV vs fluoxetine, *p* < 0.05; APV vs APV with fluoxetine, *p* < 0.01). These results suggest that the role of the NMDAR in midline crossing interacts with previously described mechanisms regulating midline axon crossing. Further, this suggests that hypoxia’s effects on midline crossing are caused in part by a reduction in NMDAR expression.

### NMDAR CRISPR knock-down and pathfinding

**Figure 3.**
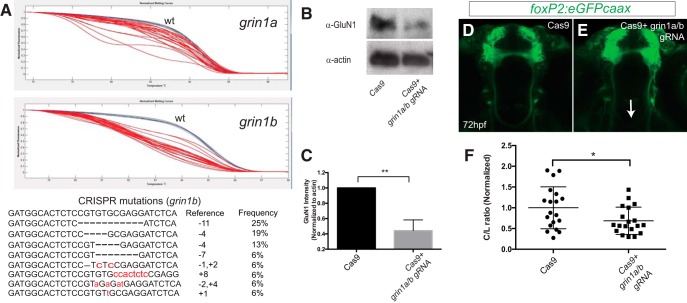
CRISPR knock-down of *grin1a/b* reduces TCPT crossing. ***A***, top panels, Normalized melt curves of PCR amplicons from individual CRISPR-injected embryos. Red melt curves show significant difference from wt curves; blue melt curves, wt controls; gray melt curves, experimental (injected) not different from wt. *Y*-axis, normalized fluorescence; *x*-axis, temperature. Bottom panel, Sequences of clones recovered from PCR amplicons at the *grin1b* locus following CRISPR mutagenesis. ***B***, ***C***, GluN1 levels (α-NMDAR) are decreased following CRISPR knock-down. Whole-animal Western blotting at 72 hpf and quantification. Triplicate, *n* > 5 embryos each experiment; **p < 0.01, Student’s *t* test. Error bars, SEM. ***D***, ***E***, Tg(*foxP2-enhancerA.2:egfp-caax*) embryos, maximum intensity z-stack projections, α-GFP immunohistochemistry, rostral top; scale bar = 50 µm. TCPTc midline axon crossing is disrupted (arrow) when treated with gRNA (***E***) compared to control (Cas9 protein alone; ***D***). ***F***, CRISPR quantification, scatterplot C/L results for TCPT crossing; *p < 0.05; Student’s *t* test.

Next, we evaluated whether knock-down of *grin1a* and *grin1b* using CRISPR led to reduced TCPTc crossing. After CRISPR knock-down of *grin1a* and *grin1b*, PCR amplification followed by HRMA showed loss of the wild-type alleles for *grin1a* and *grin1b*; sequencing of 16 PCR amplicons from the *grin1a* and *grin1b* locus showed a >80% mutagenesis rate at the target sites (Fig. 3*A*). Examples of the mutations at the *grin1b* locus show out of frame deletions or insertions in 14/16 clones (Fig. 3*A*); 2/16 clones were larger deletions or rearrangements in which the 5’ end could not be identified. With CRISPR knock-down we observed a >50% reduction in GluN1 expression on a Western blotting (Fig. 3*B*,*C*). CRISPR *grin1a/b* knock-down led to visibly and quantifiably less crossing (Fig. 3*D–F*). These results show that *grin1a* and *grin1b* mediate the function of midline crossing for the NMDAR.

### NMDAR regulation of the midline crossing decision is neuronal activity dependent

**Figure 4.**
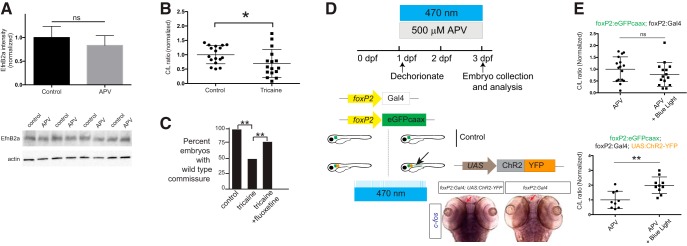
The midline crossing decision is regulated by neuronal activity controlled by the NMDAR. ***A***, EphrinB2a (EfnB2a) levels are unchanged in the telencephalon with APV treatment. Bar graphs show confocal quantification in telencephalon; ns, not significant; Student’s *t* test. Error bars, SEM. Western blotting of the four separate experiments with actin control is shown below. ***B***, Tricaine quantification, scatterplot C/L results for TCPT crossing; *p < 0.05; Student’s *t* test. ***C***, Fluoxetine rescues effects of tricaine on TCPTc. Intensity ratio data shown with values normalized to controls as bar graphs; *p < 0.05; Student’s *t* test; error bars, SEM. ***D***, Schematic workflow of optogenetic analysis. Embryos were dechorionated and exposed to APV and 470-nm light exposure from 1 to 3 dpf. Embryos were double-transgenic for *foxP2:egfp-caax; foxP2:Gal4*. Some embryos carried *UAS:ChR2-YFP* (determined by *post hoc* genotyping). If neuronal activity could rescue pathfinding, only triple-transgenic embryos would have normal midline pathfinding (arrow). Bottom pictures, *In situ c-fos* analysis of experimental embryos after 470-nm light exposure. Embryos carrying *ChR2-YFP* have increased *c-fos* expression (red arrow in region of *foxP2.A.2* neurons). ***E***, Results of optogenetic experiment show that specific restoration of activity in *foxP2.A.2* neurons is sufficient to rescue APV inhibition. Top plot, Double-transgenic embryos (no channelrhodopsin) have no rescue. Bottom plot, Triple-transgenic embryos restore midline pathfinding. C/L scatterplot results; ns, not significant; **p < 0.01; Student’s *t* test.

The NMDAR can affect cellular processes through several mechanisms, including second messenger systems and activity dependent mechanisms (Papadia and Hardingham, 2007; Kilb et al., 2011; Zhang-Hooks et al., 2016). We had found previously that midline axon crossing of the TCPTc requires down-regulation of ephrinB2a (Stevenson et al., 2012; Xing et al., 2015), and eph/ephrins have been shown to have functional effects on NMDAR function (Dalva et al., 2000; Takasu et al., 2002; Nolt et al., 2011). We tested whether NMDAR regulated ephrinB2a levels. Following APV treatment, we did not find any changes in ephrinB2a protein levels in the telencephalon (Fig. 4*A*), using Western blotting with four separate experiments of protein extracts derived from embryo heads. This argues against ephrinB2a mediating NMDAR’s effect on pathfinding. However, inhibition of neuronal activity, using the sodium channel blocker tricaine (MS-222) (Ramlochansingh et al., 2014), reduced TCPT midline crossing (Fig. 4*B*). Further, similar to the observed interaction between APV blockade and rescue with fluoxetine, we found that fluoxetine could rescue the TCPT crossing defect caused by tricaine treatment (Fig. 4*C*). These results indirectly suggest that the NMDAR could act to control the midline pathfinding decision through an activity-dependent mechanism.

To demonstrate that the NMDAR was regulating activity levels; and to test the cell-type specificity of NMDAR action, we conducted a series of optogenetic experiments (Fig. 4*D*,*E*). Embryos were dechorionated and exposed to APV and 470-nm light exposure from 1 to 3 dpf. All embryos were double-transgenic for Tg(*foxP2.A.2:egfp-caax*); Tg(*foxP2.A.2:Gal4-VP16*). Some embryos carried Channelrhodopsin2 under the inducible control of a UAS promoter, Tg(*UAS:ChR2-YFP*), which was determined by *post hoc* genotyping. If neuronal activity could rescue pathfinding, only triple-transgenic embryos would have normal midline pathfinding in the presence of APV. We found that triple-transgenic, but not double-transgenic embryos, had normal midline TCPT crossing. *in situ* expression analysis *c-fos* of experimental embryos following 470-nm light exposure showed that embryos carrying *ChR2-YFP* had increased neuronal activity, indicated by increased *c-fos* expression (red arrow in region of *foxP2.A.2* neurons; Fig. 4*D*). These results show that the NMDAR is acting by regulating neuronal activity; and that the activity is required specifically in the *foxP2.A.2* neurons.

### Arx is targeted by NMDAR

**Figure 5.**
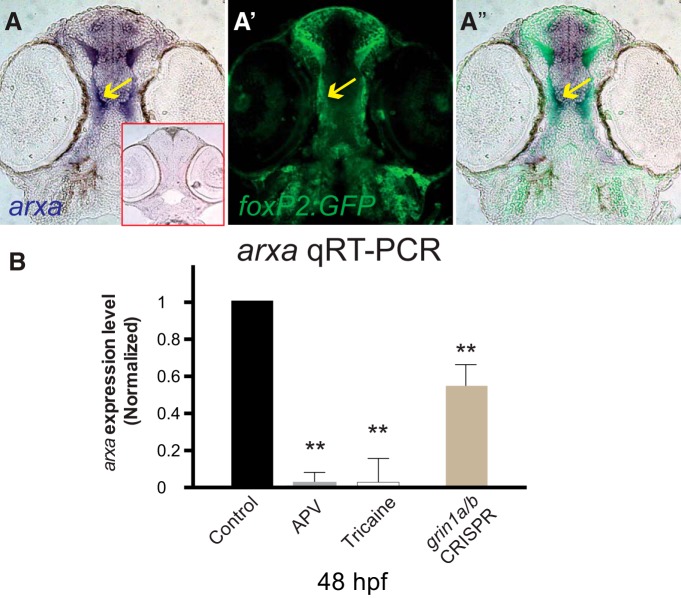
*arxa* levels are regulated by activity and the NMDAR. ***A–A’’***, *foxP2.A.2:egfp-caax* and *arxa* are coexpressed in early development as the TCPT axons extend toward the midline. Sections, rostral top, of *arxa in situ* expression and Tg(*foxP2.A.2:egfp-caax*) α-GFP immunohistochemistry. Red boxed inset shows sense probe control for *arxa*. ***B***, *arxa* expression is reduced following blockade (APV) or knock-down (CRISPR) of NMDAR or inhibiting activity (tricaine). qRT-PCR at 48 hpf, experimental triplicates; *n* > 10 embryos each group. Relative log2 fold change calculated relative to wild-type, *elf1a* standard, SD for each in parenthesis, *p* values adjusted using Benjamini–Hochberg false discovery rate; **p < 0.01; error bars, SEM.

To investigate how the NMDAR could affect pathfinding, we explored downstream effectors of axon pathfinding. As mentioned above we did not find differences in EphrinB2 expression with NMDAR blockade. We tested effects on the transcription factor *arxa* (the zebrafish ortholog of mammalian *Arx*), which has been shown by mouse knock-outs and in human genetic conditions to be necessary for normal midline commissure formation (Shoubridge et al., 2010; Simonet et al., 2015). We found coexpression of *arxa* in *foxP2.A.2* neurons during early development (Fig. 5*A*). To determine if *arxa* might be responsive to changes in NMDAR-modulated neuronal activity, we measured *arxa* expression after pharmacological blockade or genetic knock-down of the NMDAR. We performed qRT-PCR at 48 hpf in experimental triplicates, with *n* > 10 embryos in each group (Fig. 5*B*). The relative log2 fold change were calculated relative to wild type, using 28S rRNA standard, SD for each in parenthesis, with *p* values adjusted using Benjamini–Hochberg false discovery rate. We found significant decreases (*p* < 0.01) in *arxa* expression with APV or tricaine exposure, or with *grin1a/b* knock-down: respective log2 fold changes were 0.028 (0.023), 0.068 (0.037), and 0.54 (0.15). These results suggest that *arxa* might act downstream of the NMDAR to affect changes in midline crossing.

## Discussion

Midline axon pathfinding is a critical process in the development of CNS circuitry, and our results indicate an early activity-dependent step regulated through the NMDAR. To our knowledge this is the first demonstration that the NMDAR is involved in control of axon pathfinding. Interestingly, our results show that it is by regulating neuronal activity that the NMDAR affects the midline crossing decision, and potentially by targeting the transcription factor gene *arxa*.

NMDARs are known to play other roles in neuronal development including neuronal survival, dendritic and axonal arborization, and synapse formation (Ewald and Cline, 2009; Gambrill and Barria, 2011; Chakraborty et al., 2017). Also, NMDAR expression has been shown at axon growth cones (Wang et al., 2011; Gill et al., 2015) but no functional role had been shown previously.

The classical model of vertebrate axon connectivity development consisted of a relatively discrete two-step process (Katz and Shatz, 1996). This was defined by an initial stage that was genetically deterministic, leading to the development of a scaffold of axon tracts. Second, an activity-dependent stage then refined axon connections and synapse development. In invertebrates such as *Caenorhabditis elegans* and *Drosophila*, the early genetic regulation of midline commissure formation appears relatively hard-wired, with stereotypy of each neuron and its projection(s) (Sánchez-Soriano et al., 2007; Chisholm et al., 2016).

However, recent data have revealed limitations of the classical model for understanding vertebrate axon pathfinding. First, the significant variability in vertebrate axon tracts is not consistent with genetic hard-wiring (Lu et al., 2009; Mueller et al., 2013; Finn et al., 2015; Morgan et al., 2016). Second, it is now apparent that transcriptional, translational, and post-translational mechanisms all contribute to regulation of pathfinding choices (Stoeckli, 2017). Third, multiple studies have shown roles for spontaneous activity in guidance decisions earlier than had been realized (for reviewed, see Kirkby et al., 2013). This includes data showing that pathfinding decisions require rhythmic bursting neuronal activity (Hanson and Landmesser, 2004; Mizuno et al., 2007; Menelaou et al., 2008; Wang et al., 2009; Dhande et al., 2011; Plazas et al., 2013; Burbridge et al., 2014); and that corpus callosum pathfinding across the midline requires balanced hemispheric activity (Suárez et al., 2014). The spontaneous activity was shown to regulate axon guidance receptor expression, including that of EphA4, EphB1, and PlexinA3 (Hanson and Landmesser, 2004; Kastanenka and Landmesser, 2010; Plazas et al., 2013). Whether the activity directly or indirectly regulates the axon guidance receptors was not examined. Finally, environmental, non-genetic factors affect final axon trajectories and overall connectivity (Brun et al., 2009).

Our data point to an early role for neuronal activity in midline axon crossing mediated by the NMDAR. We found early expression of the NMDAR on midline crossing axons, and inhibition (pharmacologic) or reduction (hypoxia, CRISPR) of the NMDAR disrupted crossing. Using precise optogenetic stimulation, we could rescue NMDAR blockade, suggesting that the NMDAR-dependent pathfinding was regulated by activity.

We showed that the transcription factor *arxa*, a known regulator of downstream guidance receptors and a gene implicated in human midline crossing defects (Shoubridge et al., 2010; Friocourt and Parnavelas, 2011; Simonet et al., 2015), was down-regulated by NMDAR inhibition or knock-down. Interestingly, we observed that both APV and tricaine cause a greater decrease in *arxa* levels than *grin1a/b* CRISPR knock-down. A possible reason for this difference in effect is that the CRISPR knock-down targets exon 1 in the *grin1a* and *grin1b* subunits, possibly there is residual NMDAR activity by incomplete knock-down or alternative splicing (Bai and Hoffman, 2009), the former is consistent with our Western blottings showing that there is still protein expression. In contrast, APV or tricaine block any signaling or activity from the NMDAR.

In summary, these experiments provide evidence for an early activity-dependent step in midline axon pathfinding and provide a possible mechanistic insight into the regulation of activity by the NMDAR and a downstream effector via *arxa*. Understanding a potential link between activity regulated through the NMDAR, and axon pathfinding, may reveal unexpected insights into a range of neurodevelopmental connectivity disorders. For example, premature birth, which exposes the developing nervous system to chronic hypoxia (Martin et al., 2011), is associated with a range of disturbances in connectivity development even in the absence of destructive lesions (Kwon et al., 2016; Thomason et al., 2017).

10.1523/ENEURO.0389-17.2018.f1-1 Extended Data Figure 1-1 C/L ratio results for control and APV-treated embryos, including raw and normalized data. Download Figure 1-1, XLSX file.

10.1523/ENEURO.0389-17.2018.f1-2 Extended Data Figure 1-2 C/L ratio results for control and MK-801-treated embryos, including raw and normalized data. Download Figure 1-2, XLSX file.

## Synthesis

Reviewing Editor: Robert Kalb, Northwestern University Decisions are customarily a result of the Reviewing Editor and the peer reviewers coming together and discussing their recommendations until a consensus is reached. When revisions are invited, a fact-based synthesis statement explaining their decision and outlining what is needed to prepare a revision will be listed below. The following reviewer(s) agreed to reveal their identity: Martin Meyer

The authors have revised the ms. and the reviewers are satisfied
